# Efficacy, Compliance and Reasons for Refusal of Postoperative Chemotherapy for Elderly Patients with Colorectal Cancer: A Retrospective Chart Review and Telephone Patient Questionnaire

**DOI:** 10.1371/journal.pone.0055494

**Published:** 2013-02-22

**Authors:** Pan Li, Fen Li, Yujing Fang, Desen Wan, Zhizhong Pan, Gong Chen, Gang Ma

**Affiliations:** 1 Department of Colorectal Surgery, Sun Yat-sen University Cancer Center, Guangzhou, China; 2 Department of Occupational and Environmental Health School of Public Health Tongji Medical College, Huazhong University of Science and Technology, Wuhan, China; 3 Department of Intensive Care Unit, Sun Yat-sen University Cancer Center, Guangzhou, China; The University of Texas M. D. Anderson Cancer Center, United States of America

## Abstract

**Background:**

Numerous clinical trials have demonstrated that elderly patients with colorectal cancer (CRC) can benefit from chemotherapy, yet compliance in real-world practice is low. The purpose of this study is to investigate the efficacy, compliance and reasons for refusal of postoperative chemotherapy for elderly patients with CRC and to provide corresponding strategies.

**Patients and methods:**

The clinico-pathological and biochemical data of the chemotherapy group and chemo-refusing group were compared among 386 elderly patients (>70 years old) with CRC who underwent surgery. 226 patients received chemotherapy and 160 patients refused. Follow-up of the subjective reasons for refusal was investigated using the elderly caner patients' chemo-refusal reason questionnaire (ECPCRRQ) prepared by the authors and a group of psychologists. The questionnaire is administrated by telephone. A predictive model for 5-year disease-free survival (DFS) and 5-year overall survival (OS) was constructed by using Kaplan-Meier analysis, logistic and Cox regression.

**Results:**

Among stage III patients, receiving chemotherapy was associated with a significantly higher OS (68%) compared to those who refused (***OS***50%) (HR: 2.05, 95%CI: 1.12–3.77, *P* = 0.02). The Chemo-refusal group had more female and elderly patients, significantly higher rate of severe complications, and lower body mass index (BMI). Follow-up phone questionnaire analysis showed the doctors’ uncertainty of chemotherapy benefit, economic difficulties, uncomfortable feeling, superstition of Traditional Chinese Medicine, concealing information and lack of social support were the main factors for elderly CRC patients to decline chemotherapy.

**Conclusion:**

The receipt of post-operative chemotherapy in elderly patients with resected stage III CRC was associated with a more favorable survival. The low compliance rate (160/386) of postoperative chemotherapy was influenced by various subjective and objective factors.

## Introduction

Since 1990, chemotherapy has been the standard postoperative treatment for patients with stage IIB, III, IV CRC [1], [2], [3], but the efficacy and toxicity of chemotherapy in patients older than 70 years of age has been a matter of controversy. The underrepresentation of older adults in clinical trials has also been a long-standing issue resulting in limited generalizability to actual practice.and many elderly patients do not receive what is considered standard chemotherapy [4], [5], [6], [7], [8].

However, numerous studies have shown age is not a contraindication to chemotherap. With appropriate caution, older individuals may benefit from chemotherapy to the same extent as younger patients. Pooled analyses of the safety and efficacy of chemotherapy in the elderly have shown comparable toxicity rates and similar survival benefits compared with younger patients. However, these elderly patients were all eligible for clinical trial enrollment and were known to have a more favorable performance status and fewer comorbidities compared with the general elderly population [9], [10], [11], [12], [13], [14].

Subsequent studies have suggested that elderly patients were less likely to have received adjuvant therapy. In usual clinical practice, approximately 1/3 of elderly patients who had an indication for chemotherapy actually refused [7], [8], [15]. Recent calls for changes in policy have highlighted the multifactorial causes of this problem.

As the life expectancy has been increasing, elderly patients represent a growing proportion of patients with with CRC. Understanding the potential benefits, compliance and refusal reasons of postoperative chemotherapy in the elderly and providing relevant approaches will be an important tool to aid in clinician’s daily practice and decision-making.

## Methods

### Patients

This retrospective study enrolled 386 CRC(stage IIB, III, IV) patients older than 70 years who received surgical treatment in Sun Yat-sen University Cancer Center from January 2000 to January 2010. All patients were diagnosed by pathological examination and didn’t involve in neoadjuvant chemotherapy or abdominopelvic radiotherapy. Other exclusion criteria were multiple primary cancer, relapsing or dying within one month. 226 cases (58.5%) received postoperative chemotherapy and 160 cases (41.5%) refused (1.41∶1). There were three age groups: 70–74(54.1%), 75–79(33.2%), and 80–95years (12.7%).Our upper age limit was 95 years to allow for appropriate analytic cell size and to preserve anonymity. Postoperative chemotherapy regimens included 5-FU/CF, oral drugs (like UFT, XELODA), FOLFOX, XELOX and FOLFIRI. Lab tests were performed about 0.5–1 month after operation. Those cases receiving less than 3 cycles were included in the chemo-refusal group. The basic information of patients can be seen in table 1.

**Table 1:** Clinicopathological characteristics of patients.

Characteristics	Chemotherapy		*P* value
	Receiving (N/%)	Refusal (N/%)	
**Age**			<0.001
**70–74**	143(63.3)	66 (41.2)	
**75–79**	67(29.6)	61 (38.2)	
**80–95**	16(7.1)	33 (20.6)	
**Gender**			<0.001
**Male**	165 (73.0)	87 (54.4)	
**Female**	61 (27.0)	73 (45.6)	
**Location**			0.081
**Rectum**	104(46.1)	76 (47.5)	
**Sigmoid**	56 (24.8)	38 (23.7)	
**Descending**	11 (4.8)	7 (4.4)	
**Transverse**	11 (4.8)	11 (4.8)	
**Ascending**	44 (19.5)	28 (17.5)	
**Pathology grade**			0.712
**G1**	10 (4.5)	5 (3.2)	
**G2**	178 (78.7)	128 (80.0)	
**G3**	38 (16.8)	27 (16.8)	
**Stage**			0.333
**II B**	87 (38.5)	80 (50.0)	
**III**	84 (37.1)	48 (30.0)	
**VI**	55 (24.4)	32 (20.0)	
**Regimen**			<0.001
**5-FU/LV**	19 (8.4)		
**Oral drug**	90 (40.0)		
**FOLFOX**	45 (19.9)		
**XELOX**	67 (29.6)		
**FOLFIRI**	5 (2.1)		
**Living**			0.003
**YES**	157 (69.5)	99 (61.8)	
**NO**	69 (30.5)	61 (38.2)	
**Complication**			<0.001
**Yes**	13 (5.8)	35 (21.8)	
**No**	213 (94.2)	125 (78.2)	
**BSA**			<0.001
**Mean**	1.59	1.48	
**BMI**			0.004
**Mean**	26.59	20.96	

Oral drug: xeloda/UFT; FOLFOX: 5-FU/LV+oxaliplatin; XELOX: xeloda+oxaliplatin; FOLFIRI: 5-FU/LV+irinotecan; LV = leucovorin.

### Study Design

**Figure 1:**
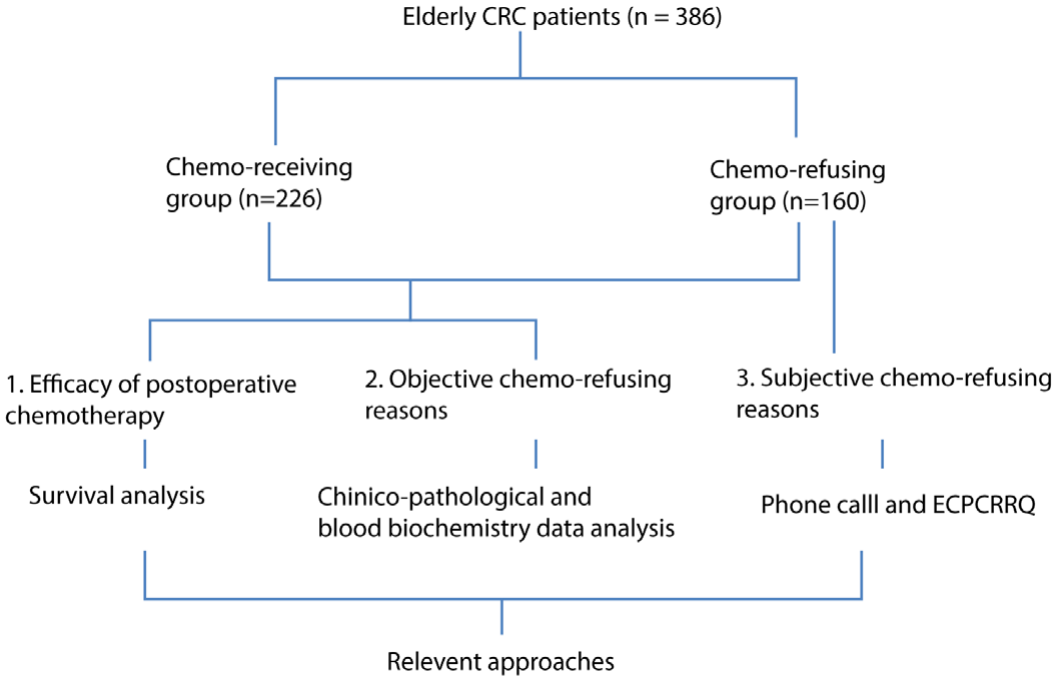
The chart of study design.

The study was perfomed at Sun Yat-sen University Cancer Center following approval by the ethic committee of Yat-sen University Cancer Center. When we reported this program to the ethic committee, we are told that it's not necessary to get the patients signing the ICF, because according to the current Chines medical regulations, the process of completing the questionnaire is non-invasive, and does not have patients' benefit hurt. We excluded the patients who does not give oral agreement via phone-call, actually we did get the oral agreement of the patient when researcher have a phone call to him/her, so no more information will be taken for patients without oral permission.The efficacy of postoperative chemotherapy was analyzed by survival analysis. The clinico-pathological and blood biochemistry data (the first follow-up check was done within one month after surgery) between the chemotherapy group and chemo-refusal group were compared. Subjective factors for refusal were evaluated using the elderly cancer patients' chemo-refusal reason questionnaire (ECPCRRQ) (table 2)prepared by the authors and a group of psychologists by phone call (as the chemo-refusal patients usually had poor visiting rates, personal interviews were not feasible). Among all 160 patients in this group, 158 questionnaires were answered by the patients or family members or both who agreed verbally to participate in the study, and their conversations were recorded. Their opinions on the reasons for chemo-refusal were assessed by a series of questions. All phone calls were conducted by an oncologist and a psychologist, and verified by another two psychologist to explore the main subjective chemo-refusing reasons. According to ECPCRRQ and the record of phone call, ten subjective chemo-refusing reasons were summarized and shown in table 3. (A refusing reason was summarized for a patient). Discordant cases were rechecked by an additional psychologist to reach a consensus. The chart of study design is shown in figure 1.

**Table 2:** Elderly Cancer Patients' Chemo-Refusing Reason Questionnaire (ECPCRRQ).

Questions		Conditions			
**1. Did the patient know about the disease**	known			unknown	
**2. The degree of doctor’s recommendation** **for chemotherapy**	No	Uncertainty of the benefit	Strongly recommendation for chemotherapy		
**3. The physical condition after surgery**	Very bad	Uncomfortable	Normal	Good	
**4. The psychological condition after surgery**	Desperately	Sad and negative	Sad and positive	Positive	
**5. Addressing complication**	No	Shorter than 2 months		Longer than 2 months	
**6. Economic condition**	Could afford the chemotherapy		Couldn’t afford the chemotherapy		
**7. Social support**	Yes		No		
**8. Patient’s opinion of chemotherapy**	Resisting	Fearing	Accepted		
**9. Patient’s opinion of Traditional Chinese Medicine**	Supertitious	Using adjuvantly	Untrusted		
**10. Convenient admittance**	No		Yes		
**11. Why did the patient not receive chemotherapy**		Open question			

**Table 3:** The summary of subjective chemo-refusing reasons.

1. The doctor wasn’t sure of the benefit of chemotherapy and didn’t recommend chemotherapy.
2. The patient trusted and used Traditional Chinese Medicine only.
3. Economic difficulty.
4. The patient was feeling uncomfortable but did other positive measures such as diet control.
5. Admitting difficulty.
6. Family members concealed the disease information to the patient.
7. Fearing of chemotherapy or lack of family support.
8. The patient was dealing with complication first and delayed chemotherapy.
9. The patient was feeling despair and refused treatment.
10. The patient couldn’t tolerate side effects of chemotherapy.

### Statistical Analysis

Data were reported as frequencies (percentages) or means and medians (range). Differences in distributions between the variables examined were assessed with the251658240^2^or the Fisher’s exact test. Patients who were alive at the last contact were censored at the last follow-up date. Surviving patients were censored on the last follow-up date. Median follow-up and the 95% confidence interval (CI) were calculated using the reverse Kaplan-Meier method. Survival curve was estimated with the Kaplan-Meier method and compared using the log-rank test. The DFS and OS rate at 5 years was reported according to different stages with its 95% CI. A multivariate Cox model was constructed. Multivariate Cox analysis included all relevant clinical variables, whatever their univariate Cox *P* value, namely age, gender, differentiation grade, complication, BSA, BMI. Two-sided *P* values of less than 0.05 were considered statistically significant. The subjective refusal reasons were reported descriptively.

## Results

### Benefits of Postoperative Chemotherapy

**Figure 2:**
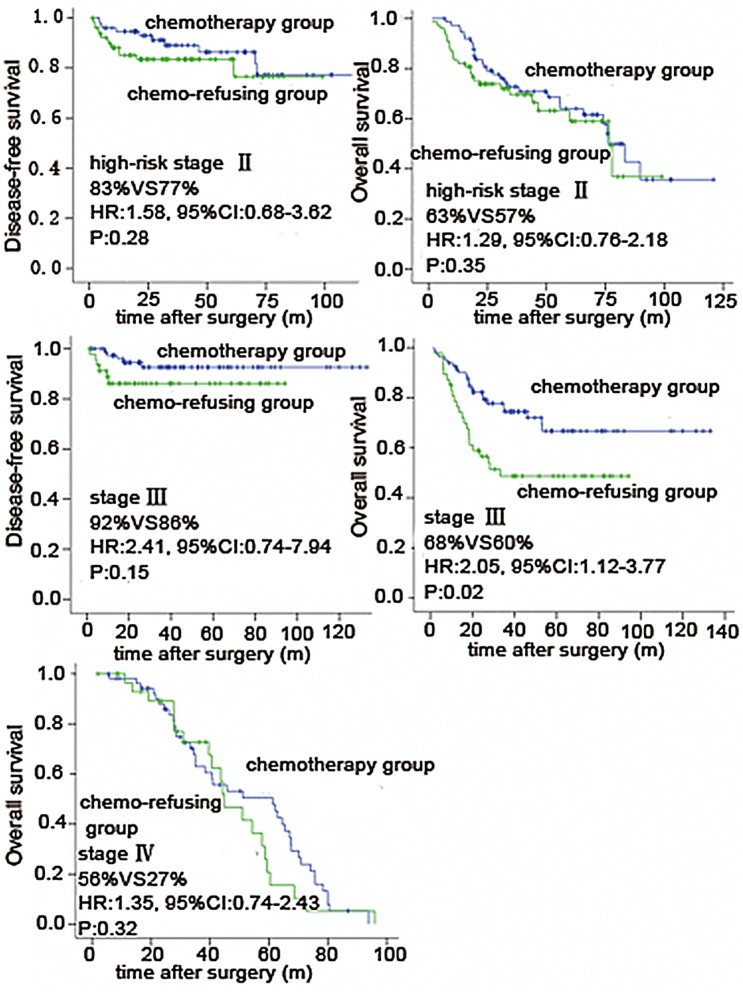
Relationship between survival and chemotherapy.

Out of all 386 cases, 41.5% of patients refused postoperative chemotherapy (226 patients received, 160 patients refused). The basic information of patients can be seen in table 1. The chemotherapy-received group showed no statistically significant improvement of 5-year disease-free survival (DFS) or overall survival (OS) in stage II B patients (DFS: 83%vs77% HR:1.58, 95%CI:0.68–3.62,*P* = 0.28; OS 63%vs57% HR:1.29, 95%CI:0.76–2.18, *P* = 0.35). There was no statistical difference of DFS between chemotherapy group and chemo-refusing group in stage III patients (92%vs86% HR:2.41, 95% CI:0.74–7.94, *P* = 0.15). But the 5-year OS was significantly higher in chemotherapy group (68%) than another group (50%)(HR: 2.05, 95%CI: 1.12–3.77, *P* = 0.02). In stage IV patients, the 5-year OS in chemotherapy group (56%) was higher than in chemo-refusing group (27%)(HR:1.35, 95%CI:0.74–2.43, *P* = 0.32). (shown in figure 2 and table 4).

**Table 4:** Relationship between survival and chemotherapy.

Stage		Chemotherapy group	Chemo-refusing group	HR	95% CI	*P* value
**II B**	5-year DFS (%)	83	77	1.58	0.68–3.62	0.28
	5-year OS (%)	63	57	1.29	0.76–2.18	0.35
**III**	5-year DFS (%)	92	86	2.41	0.74–7.94	0.15
	5-year OS (%)	68	50	2.05	1.12–3.77	0.02
**IV**	5-year OS (%)	56	27	1.35	0.74–2.43	0.32

**Table 6:** The summary of chemo-refusing reasons as shown in Table 3.

Reasons	Stage II B(N/ration)	Stage III(N/ration)	Stage IV(N/ration)
**1**	51 (63.7%)	0 (0%)	0 (0%)
**2**	10(12.5%)	3 (6.25%)	3 (9.38%)
**3**	4(5%)	9 (18.75%)	0 (0%)
**4**	2(2.5%)	9 (18.75%)	3 (9.38%)
**5**	2(2.5%)	2 (4.17%)	0 (0%)
**6**	0 (0%)	5 (10.42%)	0 (0%)
**7**	3 (3.75%)	15 (31.25%)	1 (3.13%)
**8**	7 (8.75%)	2 (4.17%)	5 (15.62%)
**9**	0 (0%)	0 (0%)	18 (56.25%)
**10**	1 (1.25%)	3 (6.25%)	2 (6.25%)
***P*** ** value**	<0.001	<0.001	<0.001

1. The doctor wasn’t sure of the benefit of chemotherapy and didn’t recommend chemotherapy.

2. The patient trusted and used Traditional Chinese Medicine only.

3. Economic difficulty.

4. The patient was feeling uncomfortable but did other positive measures such as diet control.

5. Admitting difficulty.

6. Family members concealed the disease information to the patient.

7. Fearing of chemotherapy or lack of family support.

8. The patient was dealing with complication first and delayed chemotherapy.

9. The patient was feeling despair and refused treatment.

10. The patient couldn’t tolerate side effects of chemotherapy.

### Multivariate Analyses of Objective Reasons

COX analysis showed the chemo-refusing group had more female (HR: 2.27; 95%CI: 1.48–3.48; *P*<0.001) and elderly patients (HR: 2.28; 95%CI: 1.57–3.31; *P*<0.001), significantly higher rate of severe complications (HR: 5.29; 95%CI: 2.30–12.16; *P*<0.001), low body surface area (BSA) (HR: 0.024; 95%CI: 0.003–0.02; *P*<0.001) and body mass index (BMI)(HR: 0.886; 95%CI: 0.82–0.96; *P*<0.001). There were no significant differences in tumor location, pathology, tumor staging and biochemistry tests(P<0.05) (shown in table 5).

**Table 5:** Variables in the equation.

variables	HR	95%CI		*P* value
		Lower	Upper	
**gender**	2.270	1.480	3.481	<0.001
**Age**	2.282	1.573	3.310	<0.001
**Location**	0.828	0.697	0.984	0.081
**Pathology**	0.918	0.693	1.217	0.712
**Stage**	0.808	0.585	1.118	0.333
**Complication**	5.292	2.303	12.162	<0.001
**BSA**	0.024	0.003	0.190	<0.001
**BMI**	0.886	0.815	0.962	0.004
**CEA**	0.867	0.674	1.116	0.035
**WBC**	1.313	0.119	14.446	0.883
**NE**	2.675	0.381	18.784	0.443
**HGB**	1.231	0.888	1.707	0.305
**PLT**	5.224	1.264	21.600	0.065
**ALT**	9.156	0.613	136.825	0.146
**AST**	0.166	0.009	2.931	0.192
**ALP**	4.264	1.034	17.595	0.062
**TP**	0.468	0.264	0.829	0.095
**ALB**	1.357	0.618	2.980	0.012
**TB**	3.528	0.685	18.159	0.099
**CR**	0.765	0.388	2.006	0.882

BSA: body surface area; BMI: body mass index; CEA: carcinoembryonic antigen; WBC: white blood cell; NE: neutrophils; HGB: hemoglobin; PLT: platelet; ALT: glutamic pyruvic transaminase; AST: glutamic oxalacetictransaminease; ALP: alkaline phosphatase; TP: total protein; ALB: albumin; TB: total bilirubin; CR: creatinine.

### The Relationship between Tumor Staging and Subjective Chemo-refusing Reason

Follow-up phone questionnaire analysis showed the doctors’ uncertainty of chemotherapy benefit mainly influenced the decision making of stage II patients about chemotherapy (51 cases; ratio:63.7%; P<0.001). Economic difficulties (9 cases; ratio:18.8%; P<0.001), uncomfortable feeling (9 cases; ratio:18.8%; P<0.001), and uncomfortable feeling (15 cases; ratio:31.3%; P<0.001) were the impeding factors for stage III patients. The palliative intent of chemotherapy in stage IV patients compromised the decision to receive chemotherapy (18 cases; ratio:56.3%; P<0.001). Other reasons included superstition of TCM, concealing the cancer information from the patients, and lack of family support (shown in Table 6)..

## Discussion

In clinical practice, patient age can be an influencing factor in chemotherapy decision-making. Even patients in good physical condition may place too much concern on their age. Current evidence shows that age itself is not the contraindication of chemotherapy for elderly patients [10]. Sargent DJ et al. enrolled 3351 stage III CRC patients and found adjuvant treatment had a significant positive effect on both survival and recurrence time (*P*<0.001). The five-year OS was 71% for those who received adjuvant therapy, as compared with 64% for those untreated. No significant interaction was observed between age and the efficacy of treatment, and the incidence of toxicity not significantly increased among the elderly (age>70 years) [11]. Jessup et al. assessed data from 85 934 patients with stage III CRC from 560 hospitals between 1990 and 2002, and reported that adjuvant chemotherapy increased survival in 5898 elderly patients (age>80 years) as much as it did in younger patients [12]. Folprecht et al. carried out a retrospective analysis using source data of 3825 patients who received 5-fluorouracil-containing treatment in 22 European trials and identified 629 patients with age over 70 years and found an equal overall survival in elderly patients (10.8 months) and younger patients (11.3 months, *P*  = 0.31). Response rate did not differ between age groups over 70years and less than 70 years (23.9% and 21.1%; respectively; *P* = 0.14). Progression free survival (PFS) was marginally prolonged in elderly patients (5.5 months, compared with 5.3 months, *P* = 0.01) [13]. Goldberg et al. found the relative benefit of FOLFOX4 did not differ by age for response rate, PFS (*P*  = 0.42), or OS (*P*  = 0.79) [14].

In our study, the analysis showed that stage III elderly CRC patients who received postoperative chemotherapy had a more favorable overall survival and multivariable, showed that age influenced the efficacy of postoperative chemotherapy. Follow-up by telephone questionnaires showed that the doctors, patients and family members placed importance on age in their decision-making evaluation. The proportion of elderly patients accepting chemotherapy decreased with age (*P*<0.05). This phenomenon suggests that care providers, patients and family members should receive education on the importance of establishing treatment based upon patients disease stage and physiologic status rather than chronologic age.

Older patients recovered slowly and had more complications, so physicians tend to delay chemotherapy [15]. Our data showed 72.9% of the cases with complications stopped chemotherapy. The main complications included high blood pressure, serious diabetes, myocardial infarction, stroke, epilepsy and asthma etc in our study. Phone questionnaire showed 14 cases (8.8%) stopped chemotherapy because of recovering too slowly and missing the golden time. Many clinical studies show the adjuvant chemotherapy should be commenced within 6 and 8 weeks after post surgery [16], [17], [18], [19]. The physicians deemed there was no benefit of chemotherapy if delayed more than 3 months after surgery. In the 2010 American Society of Clinical Oncology GI annual meeting, Dr. Biagi reported a meta-analysis involving 14357 cases. The results showed the mortality rate of CRC increased by 12%and recurrence rate increased by 14%with the chemotherapy delayed every month. To explain more vividly he presumed a patient: 65year old man with CRC and had no other disease, pathological report (pT3N2M0 moderately differentiated adenocarcinoma), received 5-FU chemotherapy. Survival analysis showed: no chemotherapy (5-year survival rate was 45%), if received chemotherapy within 4 weeks after post surgery (60%), within 8 weeks (55%), and within 12 weeks (50%) [15]. Therefore the doctors should not ignore those patients who delayed chemotherapy. On the other hand, the doctor must inspect the complication professionally and check if complication like high blood pressure, epilepsy etc. really influence the implementation of chemotherapy?

Among the 160 patients who did not receive chemotherapy, 16 cases (10%) received Traditional Chinese Medicine (TCM). This phenomenon was attributed to the superstition of TCM and the inadequate dissemination of information regarding the benefits of medical treatment. Even though TCM may play an important role in the effective treatment of disease, clinical studies show the OS, DFS and PFS of CRC cannot be changed by TCM alone [20], [21], [22]. Thus, physicians must clearly explain the benefits of risk of chemotherapy treatment.

Receipt of chemotherapy was associated with BSA, BMI and gender. The BMI and BSA were assessed to evaluate the nutritional status of the patients with CRC [23], [24], [25]. Male patients and those who had higher BSA and BMI tended to have positive attitude to chemotherapy (*P*<0.005). In practice, drug dose is calculated according to BSA of cases partly because BSA is relevant to body physical function (like cardiac output, liver and kidney blood flow and glomerular filtration rate) [26], [28]. A retrospective study (assessed 300 cases and 9 kinds of drug including 5-FU) concluded that BSA was not significantly related to drug clearance rate [27].Our data analysis showed that patients with low BSA recovered slowly, had poor tolerance and were more likely to have dose modifications (8 cases, median BSA: 1.43)or cessation of chemotherapy (9 cases, median BSA:1.45). Should patients with low BSA start at normal dose or reduced dose at the beginning of chemotherapy? Conversely, if a patient has no side effect of chemotherapy, should a dose-increase be considered? A new exciting discipline-therapeutic drug monitoring (TDM) is developing. The drug dose in blood can be tested during the process of chemotherapy. This method may prove to be more effective for dose individualization [29].

Several clinical trials have confirmed a survival benefit of chemotherapy for elderly patients with CRC, however metabolic differences are recognized between older and younger patients and it remains unclear whether older patients should use the same regimen of chemotherapy as their younger counterparts [30], [31], [32]. Out of the 226 cases in our series who received chemotherapy, 90 cases (39.8%) used oral drug (like 5-FU, XELODA), 45 cases (19.9%) received FOLFOX regimen, 19cases (8.4%) received5-FU/CF regimen, 67 cases (29.6%) used XELOX regimen, 5 cases (2.2%) used FOLFIRI regimen.8 cases reduced drug dose because of side-effects (3 cases FOLFOX, 5 cases XELOX), 9 cases withdrew (4 cases FOLFOX, 2 case XELOX, 1 case FOLFIRI). Other studies also demonstrate a preference for oral agents among the elderly [33], [34], [35], [36], [37], [38], [39].

There are still many other effectors and application of effective strategies required to improve the current situation such as strengthening the publicity of scientific health information and conducting routine follow-up (10% cases used TCM). The ward can also set up special chemotherapy area and simplify the admitting process. For those patients who live in remote areas, oncologists can recommend the chemotherapy regimen and guide them to receive the treatment at medical institutions closer to home (2.5% cases reported the admitting process too complex). Oncologists should also remain committed to informing patients of their true diagnosis (3.1% did not have information of their real disease). When recommending chemotherapy, consideration should be given according to the patients’ physical condition and economic status and unnecessary treatment should be avoided (8.1% cases refused because of economic difficulties). Finally, providing morale support and mobilizing all capable social support is important for better treatment and quality of life for elderly patients with CRC (11.9% cases rejected duo to expense of chemotherapy or lack of family support).

### Conclusion

The compliance rate of postoperative chemotherapy for elderly patients was low and influenced by variable subjective and objective factors which require interventions from doctors, family members and society in order to improve this situation.
